# Latent Class Analysis of Multiple Health Risk Behaviors among Australian University Students and Associations with Psychological Distress

**DOI:** 10.3390/nu13020425

**Published:** 2021-01-28

**Authors:** Melinda J. Hutchesson, Mitch J. Duncan, Stina Oftedal, Lee M. Ashton, Christopher Oldmeadow, Frances Kay-Lambkin, Megan C. Whatnall

**Affiliations:** 1 Priority Research Centre for Physical Activity and Nutrition, University of Newcastle, Callaghan, NSW 2308, Australia; melinda.hutchesson@newcastle.edu.au (M.J.H.); mitch.duncan@newcastle.edu.au (M.J.D.); stina.oftedal@newcastle.edu.au (S.O.); lee.ashton@newcastle.edu.au (L.M.A.); 2 School of Health Sciences, Faculty of Health and Medicine, University of Newcastle, Callaghan, NSW 2308, Australia; 3 School of Medicine and Public Health, Faculty of Health and Medicine, University of Newcastle, Callaghan, NSW 2308, Australia; frances.kaylambkin@newcastle.edu.au; 4 Clinical Research Design and Statistics Support Unit, Hunter Medical Research Institute, New Lambton Heights, NSW 2305, Australia; christopher.oldmeadow@hmri.org.au

**Keywords:** university students, college students, health behaviors, mental health, latent class analysis

## Abstract

University students have high rates of health risk behaviors and psychological distress. This study explores patterns of health behaviors among a sample of Australian university students, and determines whether patterns of health behaviors are associated with psychological distress and demographic characteristics. Cross-sectional data from the University of Newcastle Student Healthy Lifestyle Survey 2019 were analyzed. Fruit and vegetable intake, sugar-sweetened beverage intake, physical activity, sitting time, smoking, alcohol intake, drug use, sleep and psychological distress were assessed. Latent class analysis (LCA) was used to identify patterns of health risk behaviors, and latent class regression to explore associations between psychological distress and demographic characteristics with health behavior classes. Analysis included 1965 students (mean age 25.8 ± 8.6 years, 70.7% female). Three patterns of health behaviors were identified: healthier (48.6%), moderate (40.2%) and unhealthy (11.2%) lifestyle classes. Students in the moderate and unhealthy lifestyle classes had higher odds of moderate (OR 1.43 and 2.37) and high/very high psychological distress risk (OR 2.71 and 11.69). Students in the unhealthy and moderate lifestyle classes had a higher odds of being male, younger, enrolled in transition to university and English language courses, Aboriginal or Torres Strait Islander descent and to report some financial difficulty. Study findings may be used to inform the design of mental health interventions for university students that target key health risk behaviors.

## 1. Introduction

Attending university is an important life transition for individuals, often occurring during emerging adulthood. Successful transition to university, and academic performance can be impacted by mental health. The World Health Organization (WHO) World Mental Health International College Student (WMH-ICS) initiative generates global epidemiological data on the prevalence of mental disorders among university students [1]. Data from eight countries (19 universities) demonstrate that approximately one-third of students in their first year of study screened positive for at least one DSM-IV mental disorder (i.e., major depression, mania/hypomania, generalized anxiety disorder, panic disorder, alcohol use disorder, and substance use disorder) [2]. In addition to mental disorders, university students commonly report experiencing stress. Further analyses of WMH-ICS initiative data from nine countries (24 universities) found that most students (93.7%) indicated some perceived stress within at least one of seven life domains relevant to university students (e.g., financial situation, love life, relationship with people at work/study), and that perceived extent of stress was associated with an increased odds of mental disorders [3]. University students may also face higher rates of psychological distress than non-university students. In a sample of almost 6500 students from two Australian universities, 84% reported elevated distress levels, compared with only 29% in the general population [4].

University enrolment results in individuals facing new life experiences (e.g., different living arrangements, new social relationships, balancing study with other commitments) while also managing academic factors (e.g., class attendance, studying). These factors are known to impact student’s mental health, but they also influence students’ engagement with health risk behaviors, such as poor diet quality, physical inactivity, smoking and higher risk alcohol consumption. There is a high prevalence of health risk behaviors reported among university students globally. For example, the most recent National College Health Assessment (NCHA) surveys (2020, *n* = 75 colleges in the US, and 50,307 students) demonstrate high rates of health risk behaviors, including 68% consuming less than the recommended servings of vegetables per day, 56% not meeting physical activity guidelines, 44% sleeping less than the recommendations and 14% being current smokers [5]. Individual health risk factors are also unlikely to occur in isolation, with evidence of co-occurrence (e.g., alcohol misuse and smoking) of higher-risk behaviors and engaging in multiple higher-risk behaviors. Numerous cross-sectional and cohort studies among university students across a number of different countries have demonstrated clustering of health risk behaviors [6,7,8]. For example, an analysis of NCHA data (2010, *n* = 39 colleges, *n* = 30,093 students) via latent class analysis (LCA) identified four classes among the five behaviors explored (tobacco use, binge drinking, unhealthy diet, physical inactivity and overweight/obesity), with evidence of clustering of the health risk behaviors (i.e., high prevalence of two or more health risk behaviors) within three of the classes, which accounted for 85% of the participants [9].

Previous research has linked individual health risk factors to university student mental health, including both mental health disorders and psychological distress [10,11,12]. For example, in a cohort of students recruited at enrolment to a Canadian university (*n* = 1530), health risk behaviors at enrolment predicted positive depression screen (substance use and sleep quality) and anxiety depression screen (sleep quality) at the end of the first year of university [10]. There has, however, been limited research exploring how clusters of health risk behaviors are linked with indicators of mental health among university students [9,13,14,15]. For instance, Kwan et al. conducted a latent class analysis of eight risk behaviors (fruit and vegetable intake, alcohol use, drug use, smoking, marijuana use, sexual health, physical activity, and sleep) among Canadian university students (*n* = 837) [13]. Most students (66%) were reported to fit into the ‘typical’ class (low likelihood of smoking and illicit drugs, and low likelihood of consuming sufficient fruit and vegetables and being physically active), followed by the ‘high risk’ class (20%) (low likelihood of consuming sufficient fruit and vegetables, obtaining sufficient sleep and being physically active, and high likelihood of using illicit drugs, smoking and binge drinking). While the limited number of studies exploring associations between co-occurring risk behaviors and indicators of mental health generally demonstrate significant associations between risk behaviors and poorer mental health, they are not without limitations. Previous studies have explored only a small number of health risk behaviors and most have focused on mental health disorders as the outcome, rather than broader indicators of mental health. Finally, no studies have explored this research question among a sample of Australian university students. This is significant given the socio-cultural differences in the university environment between Australia and other countries. For example, differences exist in the rates and payment schemes for tuition fees, financial support, and typical living arrangements between Australia and other Western countries such as the USA and the UK. Many students in the USA live on campus, whereas, in Australia, living arrangements are much more variable, and factors such as these influence health behavior and outcomes.

Therefore, the aims of this study were to explore patterns of health behaviors (fruit and vegetable intake, sugar-sweetened beverage intake, physical activity, sitting time, smoking, alcohol intake, drug use and sleep) among a sample of Australian university students, and determine whether the patterns of health behaviors are associated with psychological distress and demographic characteristics. Such knowledge is imperative to the design of effective mental health promotion interventions for university students, particularly the health behavior focus, and highest-risk student groups.

## 2. Materials and Methods

### 2.1. Study Design

This study is a secondary analysis of data collected from the 2019 University of Newcastle (UON) Student Healthy Lifestyle Survey (SHLS). The SHLS is an online, cross-sectional survey aiming to identify lifestyle health risk factors (nutrition, physical activity, sitting time, sleep, alcohol intake, smoking, drug use, sexual health), mental health and overweight and obesity prevalence in UON students. The methods of the regular survey have been previously published [16]. The survey was conducted through Survey Monkey (www.surveymonkey.com.au) and allowed access on a single device to prevent multiple entries by the same individual. The survey in total included 78 questions, which were displayed across 35 pages, with an average completion time of 15 min 41 s. Questions in the survey relating to drug use, sexual health and mental health were optional to complete, where all other questions required a response. The survey was open from 9 September to 5 October 2019. These dates were chosen as they are mid-way through the second semester, and therefore do not coincide with major exams. The conduct and reporting of this work adheres to STROBE guidelines [17]. The survey included more questions than have been included within the current analysis. Data included in the current analysis were selected as indicators of each health behavior or outcome, so as not to include redundant variables in statistical models.

### 2.2. Population and Setting

All students enrolled at the UON as of 9 September 2019 (*n* = 34,924) were invited to participate. The UON is a large university with cohorts based at the main campus located in Newcastle, New South Wales (NSW), Australia and additional smaller campuses across NSW (*n* = 4), and Singapore (*n* = 1), and online/distance cohorts. Students were invited to participate via an email sent to their UON email account on the first day of the survey, with two reminder/thank you emails sent to all students on the 17 and 26 of September 2019. University of Newcastle teaching staff received an email requesting that they promote the survey in class or via the online learning management system using the recruitment materials provided by the researchers. The survey was also advertised via UON student social media accounts, digital signage across all campuses and posters at the main campus. On survey completion, participants could choose to enter a prize draw to win one of five gift vouchers valued at $AU100. All participants gave informed consent prior to completing the survey. Approval for this study was obtained from the UON Human Research Ethics Committee (H-2015-0459).

### 2.3. Measures

#### 2.3.1. Health Behaviors

Fruit and vegetable intakes were assessed using two short diet questions from the NSW Adult Population Health Survey as usual serves/day (“I don’t eat fruit (vegetables)” to “6 or more serves”) [18]. One serve of fruit was defined as 150 g and one serve of vegetables as 75 g, as per the Australian Guide to Healthy Eating (AGHE) [19]. Explanation and examples of serve sizes of fruit and vegetables including text and images were displayed alongside these questions. Fruit and vegetable intake was classified in low, moderate and higher risk categories based on Australian Dietary Guidelines as follows. Fruit intake was categorized as low intake (0–<1 serve/day), moderate intake (1 serve/day), or high/sufficient intake (at or above national recommendations of 2 serves/day). Vegetable intake was categorized as low intake (0–2 serves/day), moderate intake (3–<5 serves/day) or high/sufficient intake (at or above national recommendations of 5 serves/day). Sugar-sweetened beverages consumption was assessed using two short diet questions from the NSW Adult Population Health Survey [18], “Please indicate how many cups of (1) soft drink, cordial or sport drinks, such as lemonade or Gatorade, and (2) energy drinks, such as Red Bull or Monster, you usually drink”. Response options ranged from “≤1 cup/week” to “≥2 cups/day”, with 1 cup defined as 250 mL. The AGHE consider sugar-sweetened beverages as a discretionary choice, which therefore should only be consumed sometimes and in small amounts. Therefore, sugar-sweetened beverage consumption was categorized as low (1 cup or less/week), moderate (2–6 cups/week) or high (1 cup or more/day) consumption. Note that the consumption of diet soft drink, cordial or sports drinks was assessed as a separate question within the survey, but was not included in this analysis to avoid redundant variables in statistical models.

Physical activity was assessed via the Active Australia survey [20], including the total time (minutes) performing walking, moderate, and vigorous activity in the previous week. Total time in physical activity per week was calculated as the sum of time spent in walking, moderate and vigorous activity, with vigorous activity multiplied by two to reflect greater intensity. Participants were categorized as inactive (less than national recommendations of 150 min activity/week), active (150–300 min activity/week) or highly active (>300 min activity/week) [21].

Sitting time was assessed using questions from the NSW Adult Population Health Survey, including average time spent sitting on a weekend day and on a weekday [18]. Average total sitting time was then calculated as ((total time spent sitting on a weekday × 5) + (total time spent sitting on a weekend day × 2)/7). Participants were categorized as sitting <8 h/day, 8–11 h/day or >11 h/day, based on the evidence of greater mortality risk for each increased category of sitting time in comparison with <8 h/day [22].

Participants’ tobacco smoking status was assessed using one question from the NSW Adult Population Health Survey [18]. Those indicating that they smoke “daily” or “occasionally” were categorized as daily/occasional smokers, those indicating that they used to smoke but do not currently were categorized as past smokers, and all others as non-smokers (i.e., tried smoking but never smoked regularly, and never smoked).

Participants’ risk from alcohol intake was assessed using the Alcohol Use Disorders Identification Test (AUDIT) [23]. The AUDIT is a brief screening tool (10 items) which identifies and scores the problem use of alcohol or risk of; abstinence/low risk (0–7), moderate risk (8–15), harmful/hazardous use (16–19), or dependence (20–40).

Participants’ risk from drug use was assessed using the Drug Use Disorders Identification Test (DUDIT) [24]. The DUDIT is a brief screening tool (11 items) which identifies drug-related problems and scores as: no drug-related problems (males 0–5, females 0–1), drug-related problems (males 6–24, females 2–24), or heavily dependent on drugs (25–44).

Sleep was evaluated using one question from the National Center for Chronic Disease Prevention and Health Promotion [25]. Participants were asked to indicate their average hours of sleep in a 24-h period and were categorized as either meeting or not meeting the Sleep Health Foundation age-based recommendations (i.e., average sleep hours too short or too long) [26,27]. The recommendations are 8–10 h for 17 year olds, 7–9 h for 18–64 year olds, and 7–8 hours for those ≥65 years.

#### 2.3.2. Psychological Distress

Psychological distress risk was assessed using the Kessler Psychological Distress Scale (K-10) questionnaire [28]. The 10-item questionnaire asks participants to rate how often they had been feeling each of the ten items, (e.g., hopeless) over the previous month on a 5-point Likert scale (‘None of the time’ to ‘All of the time’). Scores for each item are summed and the level of severity of non-specific psychological distress risk categorized as: low (10–15), moderate (16–21), high (22–29), or very high (30–50).

#### 2.3.3. Socio-Demographic Factors

Demographic data collected included age, gender (male/female/non-binary/another gender identity), Aboriginal or Torres Strait Islander (ATSI) background, marital status, living situation, sources of financial support, income, ability to manage on their income, and hours of paid work, and were consistent with/adapted from the national census. Age was categorized as 17–24 years, 25–35 years, and >35 years of age. Living situation was categorized as living at their parents’ home, in their own home or other (on campus, renting, boarding/homestay or irregular). Ability to manage on their income was categorized as managing (it is easy and it is not too bad), some difficulty (it is difficult sometimes) or difficult (it is difficult all the time and it is impossible). Student-related data collected included type of degree (undergraduate, postgraduate or other) and whether they were a domestic or international student.

### 2.4. Statistical Analysis

**Figure 1:**
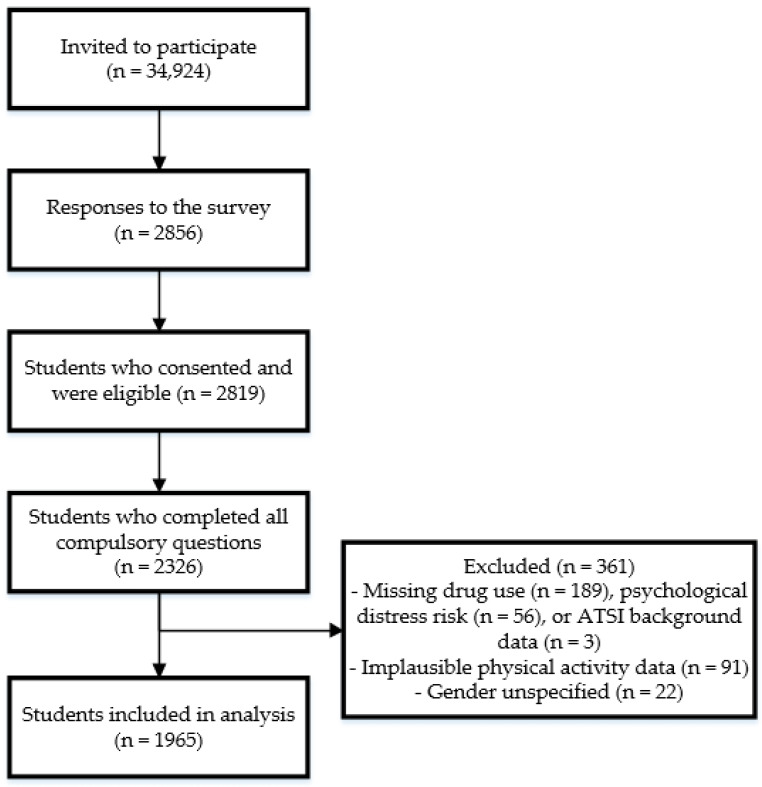
Flow of study participants.

Data were analyzed using STATA statistical software version 14.2. In total, 2819 students consented and were eligible to participate in the survey, 2326 completed the full survey and 1965 are included for this analysis (Figure 1). For this analysis, participants were excluded where they were missing data on drug use (*n* = 189), psychological distress (*n* = 56) or ATSI background (*n* = 3), due to implausible data on physical activity (*n* = 91), or where gender was specified as non-binary or another gender identity (*n* = 22). Non-binary and another gender identity participants were excluded as the sample size was too small for comparison. Health risk behaviors, psychological distress and demographic characteristics are described as number and percentage for categorical variables and mean and SD for continuous variables. Latent class analysis (LCA) was used to identify patterns of health risk behaviors. All variables were entered as categorical variables for interpretability of the findings as indicated in Table 1. Several health risk behavior variables were further categorized for the LCA due to the distribution of the data (i.e., small number of participants across some categories). Energy drinks was categorized as 1 cup or less/week, and 2–6 cups/week to 1 cup or more/day; alcohol intake risk was categorized as abstinence/low risk, moderate risk, and harmful/hazardous use or dependence; and drug use risk was categorized as no drug-related problems, and drug-related problems or heavily dependent on drugs. The outcome variable risk of psychological distress was also further categorized as low, moderate, and high or very high risk. A series of LCA models were explored specifying between two and six classes to determine the number of classes that best represented the patterns of health risk behaviors. Models were run using randomly generated seed values. The Bayesian Information Criterion (BIC) was generated for each model, where a lower BIC indicates better goodness of fit. To select the appropriate number of classes, the BIC was compared across models as well as considering the interpretability of the models. The associations between psychological distress and demographic characteristics with the health behavior classes were explored using latent class regression. Using this approach, each participant is assigned to the class for which they have the highest probability of membership, and each variable of interest (i.e., psychological distress and demographic characteristics) is added as a covariate to the model. Models were first run with each psychological distress and demographic characteristic variable individually, and a final multivariate LCA model was run including all psychological distress and demographic characteristics of interest. The association between psychological distress and demographic characteristics with the health behavior classes are reported as odds ratio and 95% confidence interval.

## 3. Results

### 3.1. Summary of Sample Characteristics

The majority of participants were female (71%), aged 17–24 years (61%) and born in Australia (83%) (Table 1). Most commonly, participants reported living in rented accommodation (39%) or their parent’s home (36%). Approximately one-third of participants (36%) reported that it was ‘difficult sometimes’ to manage on their income and 21% reported that it was ‘difficult all the time’. Most of the participants had low intake (0–<2 serves/day) of vegetables (53.6%), while just less than half (46.8%) had high/sufficient intake (2 or more serves/day) of fruit. Approximately half of the participants (52.6%) were highly physically active (>300 min/week), while the majority were meeting sleep recommendations (76.7%), sitting for <8 h/day (64.3%), and had low risk-drug use (96.5%) and alcohol use (74.3%). Twenty-seven percent of the participants were categorized as high risk of psychological distress, while 18.2% were categorized as very high risk. The median (IQR) psychological distress risk score was 20 (15–27).

### 3.2. Patterns of Health Behaviors

**Figure 2:**
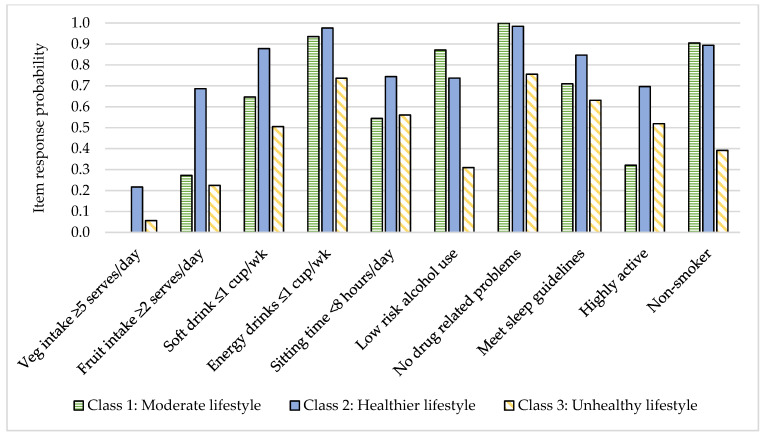
Item response probability for lowest risk category of each health behavior by health behavior class.

**Figure 3:**
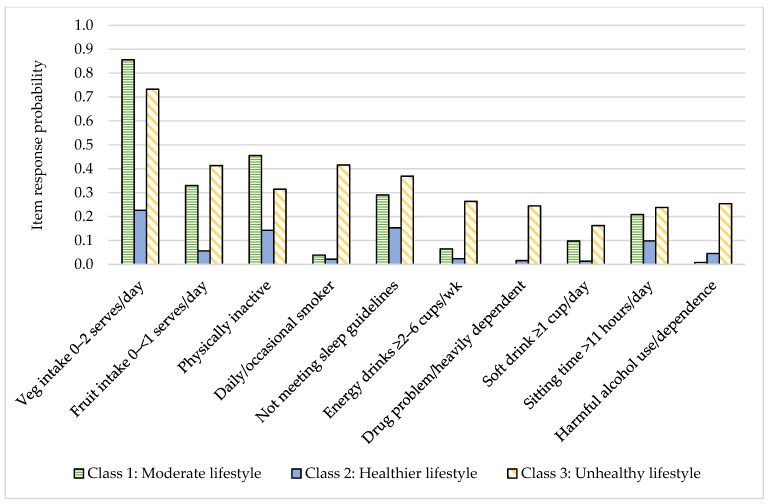
Item response probability for highest risk category of each health behavior by health behavior class.

LCA analysis identified a three-class model as the best fit and most interpretable model (Table 2). The healthier lifestyle class (48.6% of the sample) had the highest probability of consuming 5 or more vegetable serves/day, 2 or more fruit serves/day, and consuming 1 cup or less per week of soft drinks and energy drinks (Figure 2). They had the highest probability of being highly active, sitting <8 h/day and meeting guidelines for sleep, and had a high probability of low-risk drug and alcohol use. The moderate lifestyle class (40.2% of the sample) had the highest probability of consuming 0–2 vegetable serves/day and the highest probability of being inactive (<150 min/week) (Figure 3). They had the highest probability of low-risk drug and alcohol use and the highest probability of being a non-smoker. The unhealthy lifestyle class (11.2% of the sample) had the highest probability of harmful/hazardous use of alcohol or dependence, the highest probability of drug-related problems or heavy dependence on drugs, and the highest probability of being a current or past smoker. They had the highest probability of consuming soft drink and energy drinks 2–6 cups/week or more and a high probability of low fruit and vegetable intake. They also had a relatively high probability of being highly active.

### 3.3. Associations of Psychological Distress Risk and Demographic Characteristics with Health Behavior Classes

Odds of class membership for psychological distress risk and each demographic characteristic compared with the healthier lifestyle class as the referent group are presented in Table 3. Relative to those in the healthier lifestyle class, those in both the moderate and unhealthy lifestyle classes had higher odds of moderate and high/very high risk of psychological distress compared with low risk.

Relative to those in the healthier lifestyle class, those in the unhealthy lifestyle class had a higher odds of being male, younger age, enrolled in enabling (i.e., transition to university) and English language courses, and reporting some financial difficulty. Relative to those in the healthier lifestyle class, those in the moderate lifestyle class had a higher odds of having an Aboriginal or Torres Strait Islander background, and being an international student.

## 4. Discussion

This study provides new evidence into the links between dietary intake, physical activity and sedentary behavior, sleep, smoking, and alcohol and other drug use among an Australian university student sample. Three patterns of health behaviors were identified. The highest proportion (48.6%) of students fit into the healthier lifestyle class, characterized by higher probability of healthy dietary intake, being physically active, less time spent sitting, meeting sleep duration recommendations and having low-risk drug and alcohol use. The students in the moderate (40.2%) and unhealthy lifestyle (11.2%) classes had a higher likelihood of psychological distress, and were more likely to be male, younger, enrolled in transition to university and English language courses, Aboriginal or Torres Strait Islander descent and to report some financial difficulty. The findings provide further evidence of the need to do more to support university students’ to improve their health risk behaviors, as a potential strategy to manage the high levels of psychological distress. This may be of particular importance for the students in the “unhealthy lifestyle class” characterized by hazardous drug and alcohol use, smoking, higher sugar-sweetened beverage consumption and low fruit and vegetable intake, who were found to have almost 12 times greater odds of high/very high psychological distress risk.

Generally, the pattern of health behaviors in this study was that the healthier lifestyle class was characterized by healthier diet, being physically active, and obtaining sufficient sleep, and low risk substance use behaviors (alcohol, tobacco and other drugs), where the unhealthy lifestyle class was the opposite and the moderate class was mixed across most behaviors. These patterns show some similarity to studies of university student samples internationally [8,13,29], with the exception of physical activity behavior. Both the healthy and unhealthy lifestyle classes in the current study were found to have high likelihood of being physically active. However, this could be due to the overall high proportion of students that met or exceeded national physical activity recommendations. Further, the other point of difference compared with the available evidence is that the highest proportion of students in the current study fit into the healthier lifestyle class. In most studies, the majority of students have been found to report moderately healthy patterns of behavior [8,13,29]. It is important to note, however, that many of the students in this sample were not meeting national guidelines for the health behaviors assessed, and the naming of the ‘healthier’ class is therefore relative. Overall, the health behavior patterns are similar to studies of university students from other western countries, where the distinction appears to be between substance use behaviors and the other lifestyle behaviors (diet, physical activity, sedentary behavior, sleep) [8]. Therefore, different approaches may be needed in terms of supporting students with higher risk substance use, and supporting students to eat well, be more active and engage in healthy sleep practices.

The odds of having high or very high risk of psychological distress were substantially higher for those students in the unhealthy and moderate lifestyle classes relative to the healthier lifestyle class. That is, the combinations of poorer dietary intake, less physical activity, more sedentary time, poor sleep, and higher risk substance use were associated with higher risk of psychological distress. This is consistent with the findings of other studies in university student and broader adult populations, where higher risk or less healthy patterns of various health behaviors have shown to be associated with poorer mental health outcomes, including more frequent experiences of mental distress, and higher levels of psychological distress and stress [13,15,30]. There is also evidence to demonstrate the inverse. For example, Ma et al. conducted a latent class analysis among university students in Hong Kong where indicators of mental health (happiness, loneliness, life satisfaction, hopelessness, and depression) were included in the latent class models, and explored the associations of mental health classes with other health behaviors [31]. The least mentally healthy class were found to have higher odds of smoking and drug use, but similar physical activity levels compared with the normative class (moderate/average on mental health indicators). Regardless of the direction of the effect between lifestyle behaviors and mental health (i.e., poor mental health causes poor lifestyle behaviors vs. poor lifestyle behaviors causes poor mental health), the evidence supports that associations exist [32,33]. Given the high prevalence of unhealthy lifestyle behaviors (including combinations of) and mental ill health in university students, there is a clear need to address students’ health and wellbeing.

The key demographics found to be associated with health behavior classes in this study were gender, age, financial situation, Indigenous background, degree level and being an international versus domestic student. Of note, students experiencing financial difficulty, those enrolled in enabling (i.e., transition to university) courses, and those of Aboriginal or Torres Strait Islander background had higher odds of being in the unhealthy and moderate lifestyle classes compared with the healthier lifestyle class. These findings are consistent with the known health disparities between individuals of higher versus lower advantage, in terms of socio-economic status and educational attainment, and between Indigenous and non-Indigenous persons [34]. While these disparities are more pronounced in other settings, for instance rural and remote communities [35], these findings suggest that there are still differences within higher education settings that need to be addressed in order to close the gap. It was also identified that males and younger students had higher odds of being in the unhealthy or moderate lifestyle classes compared with the healthier lifestyle class. This is consistent with other studies examining health behaviors among university students and comparing younger with middle and older age adults [8,36,37]. Males have been found to engage in more risk-taking behaviors compared with females, while the association with younger age is consistent with the emerging adulthood life stage where new experiences have significant influence on lifestyle behavior choices [38,39]. It is critical that these determinants of health behaviors are addressed in health promotion efforts aiming to support students’ health and wellbeing.

The main strengths of this study include the robust analysis methods used to identify patterns of health behaviors, the broad range of health behaviors assessed, the use of validated tools for assessing health behaviors and psychological distress, and the relatively large sample size. Further, this study includes an Australian sample of university students, where there is limited research available relative to other western countries. The main limitation is the cross-sectional study design as this does not allow for determination of causality or exploration of the direction of associations. Due to the cross-sectional design, the data are limited in terms of the timeframe of reference for the reported health behaviors and does not capture effects of seasonality. Further limitations to acknowledge include the use of self-report data, as well as the representativeness of the sample which may limit the generalizability of the findings. Bias in self-reporting data may have occurred, for example responding in line with social norms, which may have impacted the findings. To minimize this, validated tools were used, the survey was anonymous and questions relating to sensitive topic areas, including mental health, were optional for participants to complete. The study sample was a small proportion of the total student body (6.7%). However, this is comparable with/higher than other recent online surveys in university students using convenience sampling and is reflective of the challenges of recruitment in university student and young adult samples [40,41,42]. The sample consisted of slightly higher proportions of female, undergraduate and domestic students compared with the average across Australian universities. However, sample characteristics were otherwise consistent and these characteristics are consistent with the University of Newcastle student body [43]. The response rate and sample characteristics may limit the generalizability of the findings to other Australian university students. Given the low response rate, data are unavailable for the majority of the student body and their health behaviors, demographic characteristics and psychological distress may differ from that of the study sample. As a result of this, and the possible biases in self-report, the study findings may not completely reflect the true associations between heath behaviors, demographic characteristics and psychological distress in this group. Additionally, while a large number of health risk behaviors and demographic characteristics were included, this was not an exhaustive list, and while a validated tool was used to assess psychological distress, this is just one indicator of mental health.

The findings from this analysis support the need for further research to better understand the influence of health risk behaviors on student’s mental health. Future research should involve longitudinal studies to track changes in university students’ health risk behaviors and mental health throughout their enrolment and determine the associations of individual health behaviors with mental health over time, along with clustering of health risk behaviors and directionality of effects with mental health. This information could then be used to determine the opportune timing of interventions to support students’ health and wellbeing, as well as tailoring and targeting to the highest-risk student groups. Further, it is important that sustained systems of tracking health behaviors and outcomes are implemented in this population group particularly in light of the current COVID-19 pandemic, where day-to-day lives, including education, are changing, which is inevitably influencing health behaviors and health outcomes [44,45], the longer-term impacts of which are not yet known. In terms of practice, these findings highlight that intervention efforts aiming to support health and wellbeing are needed for university students as a whole, and especially for particular groups within the student cohort [8,37]. This includes males, younger students, Indigenous students, those experiencing financial difficulty/low socio-economic status, and international students. Regarding the health promotion strategies themselves, whether single- or multi-behavior approaches are more effective is unclear [46]. Regardless of the approach, there still needs to be consideration of the fact that health behaviors are interrelated. Additionally, separate approaches may be needed to target substance use related behaviors (e.g., alcohol, tobacco smoking and other drugs) as opposed to other lifestyle behaviors including diet, physical activity, sedentary behavior and sleep [8]. Further, strategies to support students’ health and wellbeing need to work towards creating a health-promoting environment in line with the Okanagan International Charter for Health Promoting Universities and Colleges [47]—this means strategies that are targeted towards the university environment as well as to support university students on an individual level.

## 5. Conclusions

Limited research has explored how clusters of health risk behaviors are linked with indicators of mental health among university students. This latent class analysis identified three classes of health behaviors among a sample of Australian university students, and found that those in the moderate and unhealthy lifestyle classes had a higher likelihood of psychological distress than those in the healthy lifestyle class. The findings evidence the need for more action to support university students’ to improve their health risk behaviors, as a potential strategy to manage the high levels of psychological distress.

## Figures and Tables

**Table 1:** Demographic, health behavior and psychological distress characteristics of a sample of Australian university students (*n* = 1965).

Variable	*N*	%
**Gender**		
Male	575	29.3
Female	1390	70.7
**Age (years) (Mean ± SD)**	25.8	8.6
17–24	1191	60.6
25–35	543	27.6
>35	231	11.8
**Country of birth**		
Australia	1625	82.7
Other	340	17.3
**Aboriginal or Torres Strait Islander background**		
Yes	70	3.6
No	1895	96.4
**Marital status**		
Never married	1398	71.2
Married	284	14.5
De facto	213	10.8
Separated/Divorced/Widowed	70	3.6
**Living situation**		
Own home	278	14.2
Parents’ home	700	35.6
On campus	154	7.8
Renting	775	39.4
Boarding/Homestay	36	1.8
Irregular	22	1.1
**Income (per week)**		
$1500 or more	147	7.5
$1000–$1499	106	5.4
$500–$999	423	21.5
$1–$499	930	47.3
Nil income	221	11.2
Unsure/Don’t want to answer	138	7.0
**Ability to manage on income**		
Impossible	56	2.9
Difficult all the time	411	20.9
Difficult sometimes	698	35.5
Not too bad	587	29.9
Easy	213	10.8
**Receiving financial support**		
Yes	1167	59.4
No	798	40.6
**Hours of paid work/week (mean ± SD)**	13.6	12.9
Type of degree		
Undergraduate	1487	75.7
Postgraduate	344	17.5
Other ^a^	134	6.8
**Domestic/international student**		
Domestic	1772	90.2
International	193	9.8
**Psychological distress risk**		
Low risk	507	25.8
Moderate risk	567	28.9
High risk	534	27.2
Very high risk	357	18.2
**Fruit intake**		
0–<1 serve/day	406	20.7
1 serve/day	639	32.5
2 or more serves/day	920	46.8
**Vegetable intake**		
0–2 serves/day	1054	53.6
3–<5 serves/day	691	35.2
5 or more serves/day	220	11.2
**Soft drink intake**		
1 cup or less/week	1460	74.3
2–6 cups/week	379	19.3
1 cup or more/day	126	6.4
Energy drink intake		
1 cup or less/week	1833	93.3
2–6 cups/week	97	4.9
1 cup or more/day	35	1.8
**Physical activity**		
Inactive (<150 min/week)	566	28.8
Active (150–300 min/week)	366	18.6
Highly active (>300 min/week)	1033	52.6
Smoking status		
Daily/occasional smoker	143	7.3
Past smoker	168	8.6
Non-smoker	1654	84.2
**Meeting sleep duration recommendations**		
No	457	23.3
Yes	1508	76.7
**Sitting time**		
<8 h/day	1264	64.3
8–11 h/day	390	19.9
>11 h/day	311	15.8
**AUDIT classification**		
Abstinence/low risk	1460	74.3
Moderate risk	399	20.3
Harmful/hazardous use	52	2.7
Dependence	54	2.8
**DUDIT classification**		
No drug-related problems	1896	96.5
Drug-related problems	56	2.9
Heavily dependent on drugs	13	0.7

^a^ Includes students enrolled in enabling (i.e., transition to university) courses and English language courses.

**Table 2:** Latent class analysis item response probabilities for health behaviors among a sample of Australian university students (*n* = 1965).

Health Behavior		Health Behavior Class		
		Moderate Lifestyle	Healthier Lifestyle	Unhealthy Lifestyle
Latent Class Membership (%)		40.2	48.6	11.2
Vegetable intake	0–2 serves/day	0.86	0.23	0.73
	3–<5 serves/day	0.14	0.56	0.21
	5 or more serves/day	0.00	0.22	0.06
Fruit intake	0–<1 serve/day	0.33	0.06	0.41
	1 serve/day	0.40	0.26	0.36
	2 or more serves/day	0.27	0.69	0.22
Soft drink intake	1 cup or less/week	0.65	0.88	0.51
	2–6 cups/week	0.26	0.11	0.33
	1 cup or more/day	0.10	0.01	0.16
Energy drink intake	1 cup or less/week	0.94	0.98	0.74
	2–6 cups/week to 1 cup or more/day	0.06	0.02	0.26
Physical activity level	Inactive (<150 min/week)	0.46	0.14	0.31
	Active (150–300 min/week)	0.22	0.16	0.17
	Highly active (>300 min/week)	0.32	0.70	0.52
Smoking status	Daily/occasional smoker	0.04	0.02	0.42
	Past smoker	0.06	0.08	0.19
	Non-smoker	0.90	0.89	0.39
Meeting sleep recommendations	No	0.29	0.15	0.37
	Yes	0.71	0.85	0.63
Sitting time	<8 h/day	0.54	0.74	0.56
	8–11 h/day	0.25	0.16	0.20
	>11 h/day	0.21	0.10	0.24
Alcohol use risk	Abstinence/low risk	0.87	0.74	0.31
	Moderate risk	0.12	0.22	0.44
	Harmful/hazardous use or dependence	0.01	0.05	0.25
Drug use risk	No drug-related problems	1.00	0.98	0.76
	Drug-related problems or heavily dependent on drugs	0.00	0.02	0.24

**Table 3:** Odds of class membership by psychological distress risk and demographic characteristics among a sample of Australian university students (*n* = 1965).

	Odds Ratio (95% CI)		
	Healthier Lifestyle	Moderate Lifestyle	Unhealthy Lifestyle
Psychological distress risk: Moderate ^a^	Ref	1.43 (0.96–2.12)	2.37 (1.04–5.43)
Psychological distress risk: High/very high ^a^	Ref	2.71 (1.84–4.00)	11.69 (5.47–24.97)
Age: 25–35 years ^b^	Ref	0.65 (0.36–1.17)	2.06 (0.73–5.85)
Age: 17–24 years ^b^	Ref	0.53 (0.28–0.99)	1.44 (0.47–4.45)
Gender: Male ^c^	Ref	1.15 (0.79–1.68)	5.81 (3.52–9.60)
Aboriginal or Torres Strait Islander background: Yes ^d^	Ref	2.42 (1.13–5.18)	1.63 (0.55–4.84)
Living situation: Own home ^e^	Ref	0.53 (0.30–0.92)	0.72 (0.29–1.77)
Living situation: Other ^e, f^	Ref	0.44 (0.31–0.63)	1.21 (0.72–2.01)
Type of student: International ^g^	Ref	13.06 (6.74–25.30)	1.92 (0.57–6.40)
Type of degree: Postgraduate ^h^	Ref	0.65 (0.40–1.05)	0.74 (0.36–1.54)
Type of degree: Other ^h, i^	Ref	1.55 (0.79–3.04)	5.33 (2.60–10.90)
Managing on income: Some difficulty ^j^	Ref	1.24 (0.89–1.74)	2.26 (1.29–3.97)
Managing on income: Difficulty ^j^	Ref	1.39 (0.89–2.16)	5.70 (3.19–10.22)

^a^ Compared with low psychological distress risk. ^b^ Compared with >35 years of age. ^c^ Compared with female. ^d^ Compared with non-Aboriginal or Torres Strait Islander background. ^e^ Compared with living in parents’ home. ^f^ Other living situation includes living on university campus, renting, boarding/homestay or irregular. ^g^ Compared with domestic students. ^h^ Compared with undergraduate student. ^i^ Includes students enrolled in enabling (i.e., transition to university) courses and English language courses. ^j^ Compared with managing on income.
